# A detailed characterization of peripheral blood lymphocytes in patients with myeloproliferative disease treated with pegylated-interferon alpha

**DOI:** 10.1186/2051-1426-3-S2-P258

**Published:** 2015-11-04

**Authors:** Magdalena Kovacsovics-Bankowski, Christy Warby, Olga Effimova, Todd W Kelley, Soo Kim, Sabina Swierczek, Josef Prchal

**Affiliations:** 1 University of Utah, Salt Lake City, UT, USA; 2 ARUP Institute for Clinical and Experimental Pathology, Salt Lake City, UT, USA

## 

Pegylated-Interferon alpha (Peginfa) treatment of patients with polycythemia vera or essential thrombocythemia has resulted in long-term clinical response, decrease *JAK2*^V617F^ allelic burden and restoration of polyclonal hematopoiesis.

Characterizing the peripheral blood lymphocytes (PB) repertoire allows for monitoring the effect of immune-based therapies such as Peginfa. Here we analyzed the phenotype and frequency of PB lymphocytes from Peginfa treated patients and compared them to patients treated with cytoreductive drugs.

Samples collected pre and at various time during treatment were analyzed using a multicolor flow cytometry panel containing antibodies to CD3, CD4, CD8, CD25, CD56, CD38, CD39, CD45RO, CD197, HLA-DR, PD-1, OX40, 4-1BB, CTLA-4, Foxp3, Helios and Ki-67.

We found that Peginfa increased the frequency of PB Foxp3^+^ CD4^+^ regulatory T cells (Treg) from 6.43% to 9.62% (p=0.0033). Highly suppressive Treg, characterized by co-expression of CD39 and HLA-DR were also increased in PB from Peginfa treated patients (18.92% to 22.92, p=0.0401). We also found an augmentation of cycling CD8^+^ T cells (4.6% to 7.45% (p=0.0010)) and NK from 8.54% to 17.49 % (p=0.0156) cells as measured by expression marker Ki-67.

Our results also show that Peginfa increased the frequency of PD-1^+^ CD4^+^ helper cells (p=0.0028), PD-1^+^ CD4^+^ Foxp3^+^ Treg cells (p=0.0129) and PD1^+^ CD8^+^ T cells (p=0.0391). We did not observe any changes in these lymphocyte populations in the PB of hydroxyurea treated patients.

Overall these results indicate that there is a strong stimulation of the immune response as measured in the PB that may contribute to eradication of *JAK2*^v617F^ clones. These results will be correlated with *JAK2*^v617F^ allelic burden and for female patients, with recovery of a polyclonal hematopoiesis as measured by X chromosome allelic usage. More studies need to be performed on bone marrow lymphocytes to determine the composition and function of T cells in the vicinity of *JAK2*^V617F^ positive clones.

